# Supplementary figures and images for: Dynamic neuromuscular remodeling precedes motor-unit loss in a mouse model of ALS
Source: eLife. 2018 Oct 15;7:e41973. doi: 10.7554/eLife.41973 (PMC6234026; doi:10.7554/eLife.41973)

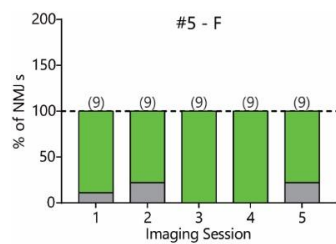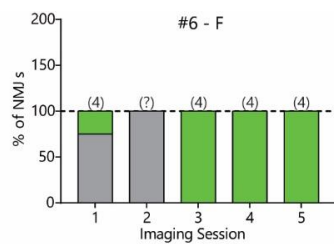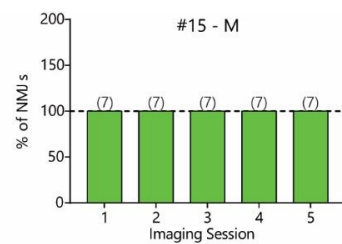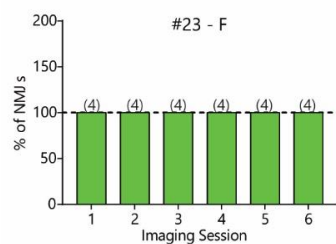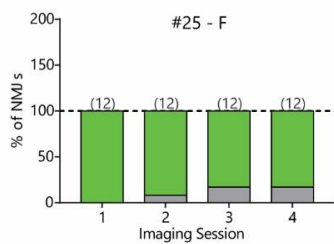

■ Fully inn. by MU  
■ Not observable NMJs  
 (x) # of NMJs inn. by MU

Supplement: Figure 1—source data 1. — In the spreadsheet, a value of ‘1’ represents an NMJ fully innervated by the MU while a value of ‘x’ represents an NMJ which could not be fully resolved on that session. The global percentages for each MU are included below each table with individual histograms for each MU. The histograms are also compiled in the PDF file. [file elife-41973-fig1-data1.zip › Figure1_source-data-1/Figure1_IndivGraphs.pdf]

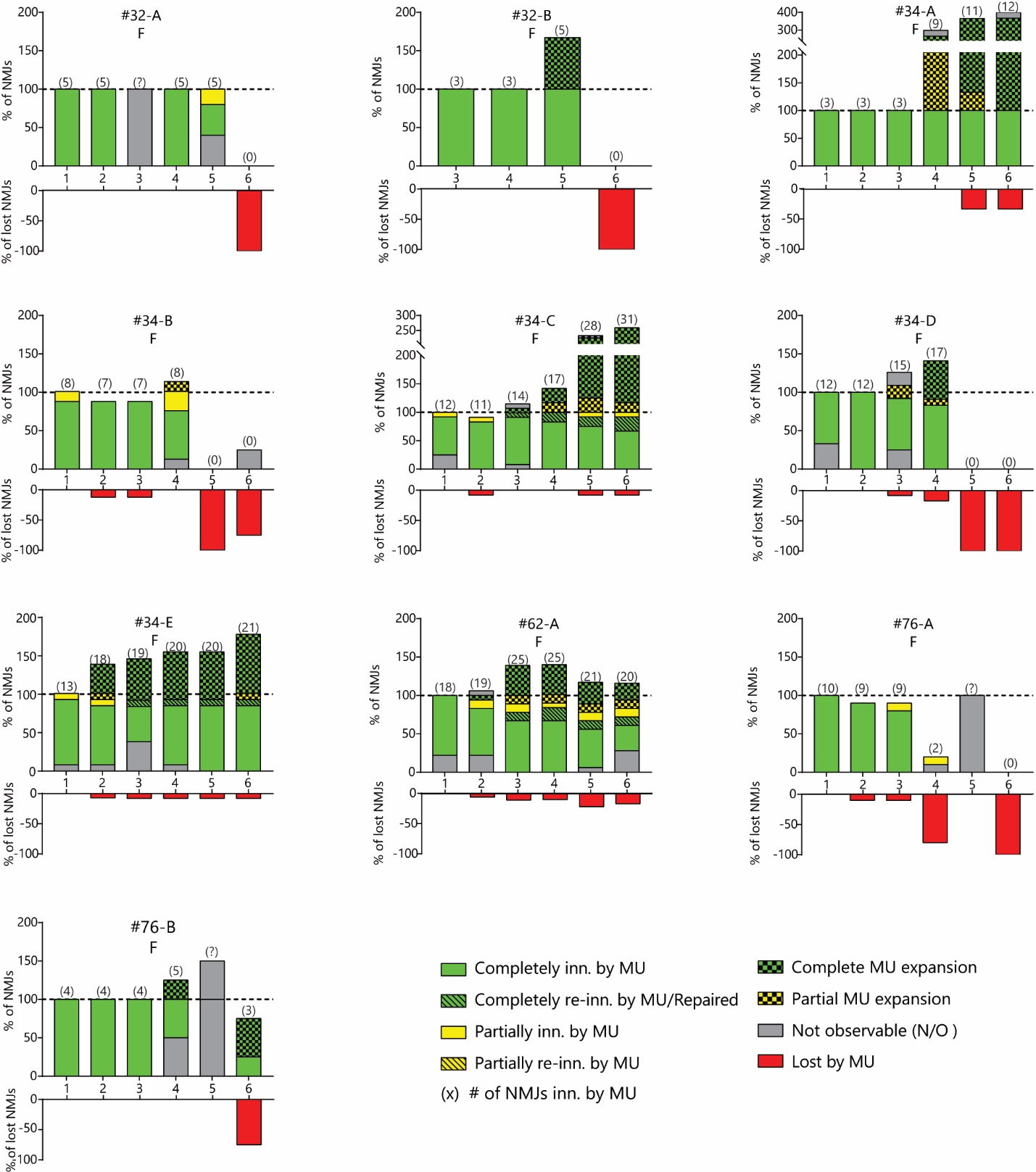


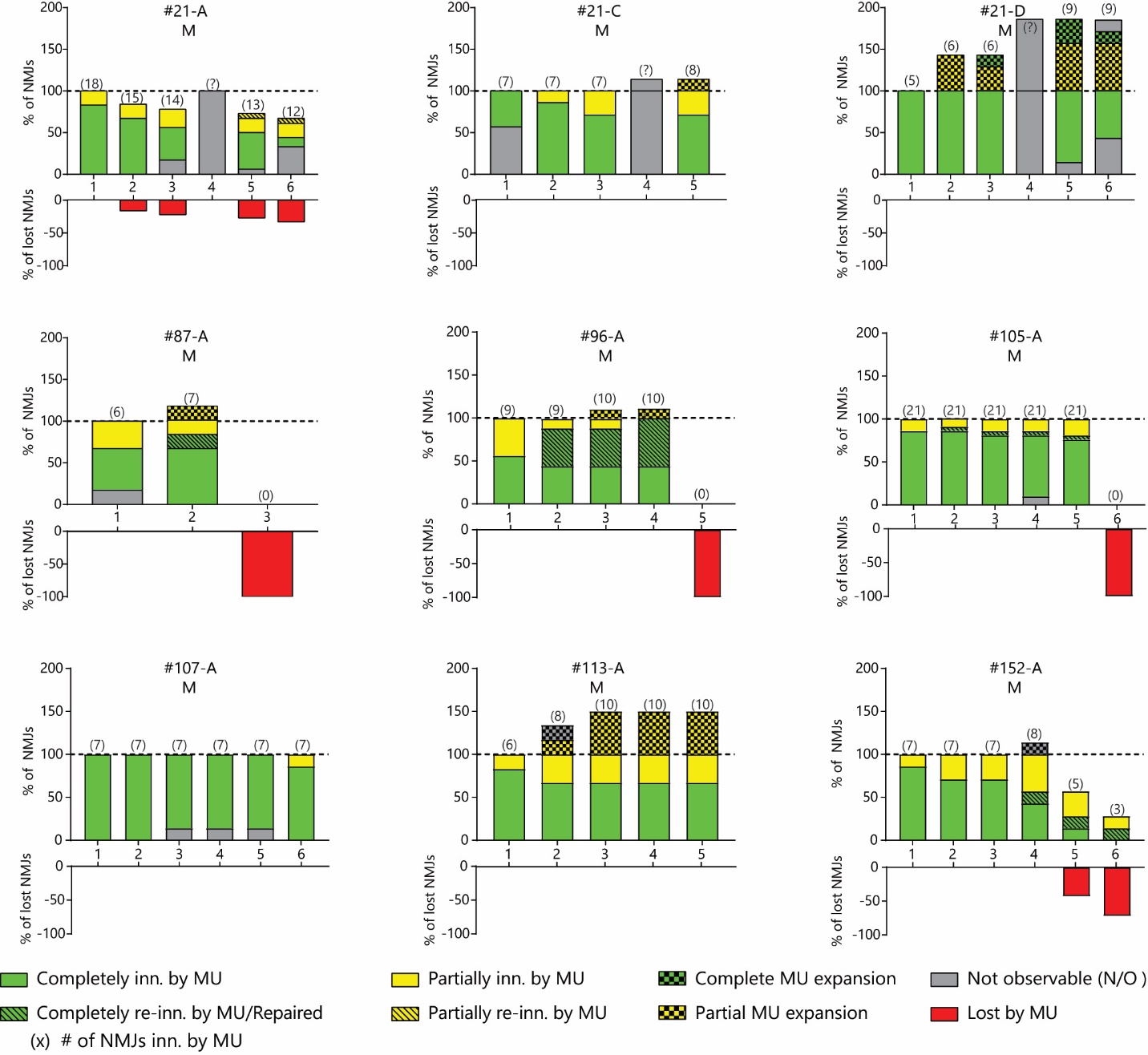

Supplement: Figure 4—source data 1. — In the spreadsheet values of ‘1’, ‘0.5’ and ‘0’ represent an NMJ which is fully innervated, partially innervated or not innervated by the imaged MU. A value of ‘x’ represents an NMJ which could not be fully resolved on that session. The change relative to last session (delta) and the classification of the NMJ (repair, reinnervated or new) of each NMJ as well as the global percentages for each MU and individual histograms for each MU are presented below the innervation status of each NMJ. The data is split between three tabs: the females, the males and the compilation, which contains the individual data points for the histogram in Figure 4C. The histograms are also compiled in the PDF file. [file elife-41973-fig4-data1.zip › Figure4_source-data-1/Figure 4_indivGraphs.docx]
